# Bridge Resistance Compensation for Noise Reduction in a Self-Balanced PHMR Sensor

**DOI:** 10.3390/s21113585

**Published:** 2021-05-21

**Authors:** Jaehoon Lee, Changyeop Jeon, Taehyeong Jeon, Proloy Taran Das, Yongho Lee, Byeonghwa Lim, CheolGi Kim

**Affiliations:** 1 Department of Emerging Materials Science, DGIST, Daegu 42988, Korea; ljh@dgist.ac.kr (J.L.); jco4268@dgist.ac.kr (C.J.); wonil075201@dgist.ac.kr (T.J.); 2 Magnetics Initiative Life Care Research Center, DGIST, Daegu 42988, Korea; proloy@dgist.ac.kr; 3 Quantum Magnetic Measurement Team, KRISS, Daejeon 34113, Korea; yhlee@kriss.re.kr

**Keywords:** magnetoresistive sensors, self-balanced bridge, planar Hall magnetoresistance, offset compensation

## Abstract

Advanced microelectromechanical system (MEMS) magnetic field sensor applications demand ultra-high detectivity down to the low magnetic fields. To enhance the detection limit of the magnetic sensor, a resistance compensator integrated self-balanced bridge type sensor was devised for low-frequency noise reduction in the frequency range of 0.5 Hz to 200 Hz. The self-balanced bridge sensor was a NiFe (10 nm)/IrMn (10 nm) bilayer structure in the framework of planar Hall magnetoresistance (PHMR) technology. The proposed resistance compensator integrated with a self-bridge sensor architecture presented a compact and cheaper alternative to marketable MEMS MR sensors, adjusting the offset voltage compensation at the wafer level, and led to substantial improvement in the sensor noise level. Moreover, the sensor noise components of electronic and magnetic origin were identified by measuring the sensor noise spectral density as a function of temperature and operating power. The lowest achievable noise in this device architecture was estimated at ~3.34 $\mathrm{nV}/\sqrt{\mathrm{Hz}}$ at 100 Hz.

## 1. Introduction

Detection of the ultra-low magnetic field has a wide area of interest where sensitive physical sensing holds the key part of applicative technologies such as automotive [1], magnetic communication [2,3,4,5,6], noninvasive brain mapping [7], nondestructive materials evaluation [8], geomagnetism [9,10], and point-of-care diagnostics [11,12,13,14]. In particular, magnetic field sensors have been applied in the engine-based automotive industry over decades to control the speed, navigation, steering, and parking modules of automatic control systems. Modern automotive accessories have been using magnetic sensors to measure power consumption accurately, and their application is extended in areas such as smart grids and power-distribution units (PDUs) [15,16,17]. In addition, the highest-resolution magnetic sensors have been used in various forms in the biomedical industry, such as in early diagnosis of diseases, health monitoring, and point-of-care diagnostics [18,19]. Based on recent development in other magnetic-field-sensing technologies, magnetoresistive (MR) sensors are the most attractive due to their room-temperature operation for 3D electronic compasses and electronic vehicles, with a small size and low cost [20,21,22,23].

Additionally, due to the demand for high-temperature stability, high-resolution field sensing, and low power consumption, several research groups have been actively working in different MR technologies over decades to develop novel sensor materials, especially in the fields of AMR (anisotropic magnetoresistance), GMR (giant magnetoresistance), TMR (tunneling magnetoresistance), and PHMR (planar Hall magnetoresistance) technologies [24,25,26,27,28,29]. To achieve very high sensitivity and ultra-low detectivity at room temperature, several works have also been reported regarding the development of sensor geometrical design, including the integration of magnetic flux concentrators (MFCs) [24,25,30,31]. Importantly, in most of these cases lock-in technology, chopper, or auto-zero amplifiers are used to reduce the sensor noise level; however, those amplifiers show a thermal drift and 1/f noise at their input [27,32,33,34]. To overcome this limitation, manipulation of the sensor’s geometrical configuration is one of the promising solutions to improve the sensitivity, detectivity, and stability of the magnetic sensor. In this perspective, the most common choice is to use two signals in differential mode [35,36] or the self-balanced bridge configuration [37,38,39]. In differential mode, the same offset voltage signal and mimic characteristic of the sensor system are primarily required to reduce the possibility of different offset voltage errors effectively. Due to the unavailability of such an ideal sensor’s fabrication, an application-specific integrated circuit (ASIC) based on a mathematically complex software algorithm is used in industry to correct the sensor output signal [40,41,42,43]. This methodical approach carries several drawbacks due to complexity in the mathematical program and the requirement of complex circuit techniques. Moreover, it does not remove the offset voltage error completely from the sensor itself, but rather converts the offset voltage to any arbitrary value and includes an additional noise in the calibration circuit, which eventually reduces the sensor functionality at a low-frequency noise level.

To overcome this problem, self-balanced bridge sensor architecture is an ideal candidate to reduce the intrinsic offset voltage of the device at the wafer level, as it exhibits no current due to resistance imbalance regardless of which sensor type is used. In ideal MR self-balanced bridge configuration, field-independent base resistances are canceled out, and the remaining field-sensitive components in each bridge branch eventually enhance the field sensitivity of the magnetometer. Here, the current direction in adjacent branches are generally 90°, thus PHMR has an advantage for bridge configuration in single-batch fabrication because the sign of resistance in each branch is given by the function of the angle between current and magnetization, rather than the angle between the magnetizations of adjacent layers, as in GMR and TMR sensors. However, it shows an intrinsic offset voltage error due to fabrication errors such as lithography errors and deposition uniformity, which leads to the generation of additional noise. Note that this additional noise can be minimized using a reconfigurable self-balanced bridge sensor with offset voltage compensation at the wafer level.

In this article, noise measurements in the frequency range of 0.5 Hz to 200 Hz are presented for a reconfigurable self-balanced bridge-type exchange-coupled PHMR sensor. Significant improvement in noise spectral density (NSD) was observed implementing a tunable offset voltage compensation method at the wafer level, which led an improvement in sensor detectivity by an order of magnitude. Furthermore, operating voltage and current dependent noise spectra were analyzed to elucidate various sensor noise contributions such as intrinsic noise of the sensor itself, extrinsic operational noise, and intermixing noise. Here, “intermixing” refers to the intercorrelation between sensor parameters and extrinsic operation power noise. The fabrication process of PHMR-based sensors is very simple, thereby facilitating a *cost*-*effective* wafer-level *fabrication of this kind of sensor. Moreover*, they can detect sensitively an ultra-low magnetic field on the order of sub-nT. Due to the lower detection limit, small size, and low power consumption, these sensors are favorable for high-sensitivity technological applications, especially in low-frequency regimes. In particular, low magnetic field sensing fields such as magnetic biosensors, high-resolution three-axis compasses, and high-resolution current sensors are the best applicative choice of PHMR for sensors whose detectivity is heavily affected by noise level.

## 2. Materials and Methods

### PHMR Sensor Fabrication and Experimental Details

**Figure 1:**
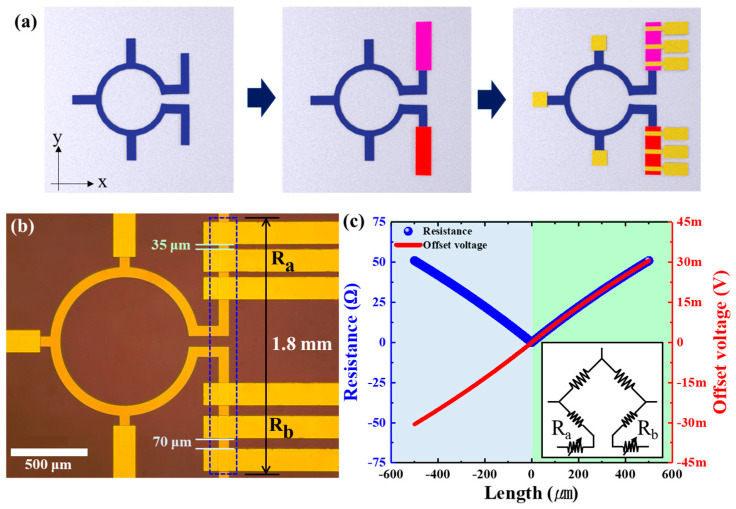
(**a**) Structure diagram of the self-balancing bridge-type PHMR sensor with resistance compensator applied. The first is the magnetic field sensing area, marked in blue. The second is a resistance compensator, marked in red and purple. The last is the electrode, marked in yellow. (**b**) Microscopic image of the fabricated sensor. The resistance compensator was manufactured to generate the offset voltage up to the maximum voltage allowed by the preamp in order to clearly confirm the ability to adjust the offset voltage. The electrode spacing of each resistance compensator $R_{a}$ and $R_{b}$ was 35 μm and 70 μm, respectively, and the resistance was 20 Ω and 40 Ω, respectively. (**c**) The change in the apparent resistance of the y-axis (blue) and the offset voltage (red) of the sensor according to the change in the length of the resistance compensator. Here, it is assumed that the length change of $R_{a}$ and $R_{b}$ did not occur at the same time. The increase in the length of $R_{a}$ was expressed as a negative number, and a blue background was used, and the increase in the length of $R_{b}$ was expressed as a positive number, and a green background was used. The inserted figure shows the circuit diagram of (**b**).

The PHMR sensor was fabricated using a bilayer magnetic thin film deposited by a DC-magnetron sputtering system. The bilayer structure was made of Ta(5 nm)/NiFe(10 nm)/IrMn(10 nm)/Ta(5 nm) and grown on a 500 nm SiO_2_ substrate using the wet oxidation process. The film was capped with a thin Ta layer (5 nm) to prevent oxidation. The bilayer structure was exchange-coupled and had negligible hysteresis for magnetic fields perpendicular to the exchange bias [44,45]. A uniform magnetic field of 25 mT was applied in the film plane during the deposition to induce the magnetic anisotropy in the ferromagnetic layer to define the exchange bias direction in the bilayer structure. During the thin film deposition process, a base pressure of 1.7 × 10^−7^ Torr was maintained under an Ar (99.999 %) pressure of 3 × 10^−3^ Torr. The ring-type self-balanced bridge sensor, the resistors, and electrode were patterned through UV–photolithography and a lift-off process to design the potentiometer circuit of the compensator and electrical contact pads. Figure 1a shows a reconfigured ring-shaped, self-balanced bridge-type sensor with a potentiometric circuit to tune the resistance of the sensor branch. The bridge architecture was designed with a five-electrode structure. The resistance tuner compensators for both arms ($R_{a}$ and $R_{b}$_)_ were marked by a blue dotted regime (see Figure 1b), and were separated by 35 μm and 70 μm, respectively, from the electrode to adjust the resistance of each branch. Figure 1c depicts the variation of sensor offset voltage as a function of variable bridge branch length from positive to negative end (red line), and the resistance compensation performance with the changes in branch length (blue line). The resistance compensator demonstrated the additional resistance adjustment functionality for both arms (see Figure 1a) in the proposed self-balanced bridge-type sensor. The detailed compensation method of the resistance compensator is explained in Appendix A.

**Figure 2:**
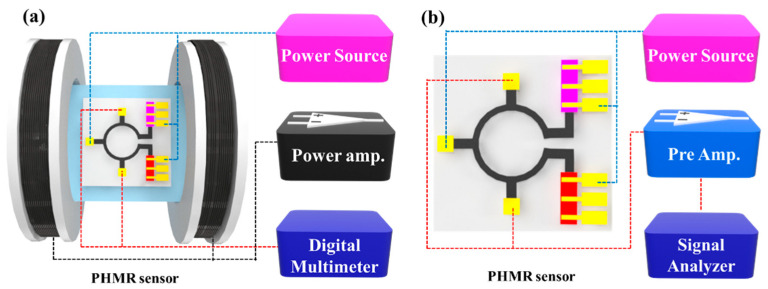
Schematic experimental setups for (**a**) PHMR sensor profile measurements and (**b**) noise spectrum density measurements.

For the sensor characterization, a 2400 Keithley source meter was used to pass the operating current/voltage to the sensor, whereas the output voltage and offset voltage were measured using an HP 34401A digital multimeter. An Agilent 35670A spectrum analyzer was used for noise evaluation. Note that for each measurement, the sensor signal was amplified using a specially designed low-noise preamplifier [46]. In this experimental environment, the amplifier exhibited a white noise level of 1 $\mathrm{nV}/\sqrt{\mathrm{Hz}}$ with a gain of 60 dB. The experimental setup for the PHMR sensor profile measurement system and the noise measurement system are shown in Figure 2a,b, respectively.

In this study, the role of three fundamental noise components, (i) intrinsic noise from sensor itself, (ii) extrinsic noise from the operating current and environment, and (iii) their intermixing noise, were analyzed as follows: First, an OTF-1200X furnace manufactured by MTI Corporation was used to measure thermal noise. For the experiment, the sensor was kept at the center horizontally inside a quartz tube furnace, and the temperature was varied from 303 K to 373 K to measure the NSDs. In this case, all measurements were performed in Ar atmosphere to prevent sensor oxidation due to the rise of temperature. Second, extrinsic noise contributions were analyzed using a simple experimental setup connecting a low-noise amplifier and a noise spectrum analyzer. Usually, this noise component originates due to deployed instruments and other environmental factors. Third, noise contributions for different sensor-driving systems, such as a Keithley 2400 source meter and battery source module (1.5 V AA battery type), were recorded and analyzed to reveal the effectiveness of source contributions in total sensor noise at constant current or voltage mode. Note that the intermixing noise was generally affected by shape, material, and thin-film structure of the sensor; however, in this study, changes in noise contribution due to reconfiguration of bridge architecture were investigated at the wafer level.

In this perspective, the ring-shaped PHMR sensor was fabricated with an unbalanced resistance in a self-balanced bridge architecture, and the correlation between sensor offset voltage and the noise level was devised by tuning the resistance bridge compensator as shown in Figure 1a. Furthermore, operating bias voltage and current-dependent noise measurements were performed to analyze the change in sensor offset voltage levels with its NSDs. Note that every noise measurement was carried out in an unshielded environment for various bias currents and voltages with a ring-type sensor with a 500 μm sensing radius and 70 μm line width.

## 3. Results

### 3.1. Noise Classification

In this classified noise model, sensor noise includes the thermal noise and 1/f noise contribution of the sensor. By taking account of the operating power source and pre-amplifier, the total effective average noise contribution of the sensor can be expressed as:

(1) $$V_{noise}=\sqrt{V_{int}{}^{2}+V_{ext}{}^{2}+V_{mix}{}^{2}}$$

where *V_int_*, *V_ext_*, and *V_mix_* are the intrinsic noise, extrinsic noise, and intermixing noise contributions, respectively. Intrinsic noise was originated by the sensor itself, including thermal and 1/*f* noise. Generally, in the low-frequency regime, the 1/*f* component was more dominant than thermal noise, whereas in the high-frequency range, the thermal or white noise contribution was more predominant. In this context, the effective average noise of the sensor was estimated at 100 Hz, where white noise was prevailing compared to its 1/*f* counterpart. It is more likely that a similar analytical approach can also be employed for noise analysis in a higher frequency range (~100 kHz) due to dominance of white noise. Thus, only the thermal noise component is considered here as a prime contributory factor for sensor intrinsic noise. In addition, the contribution of extrinsic noise, caused by noise fluctuation of the measurement system and surrounding environment, was taken into consideration, along with its intermixing counterpart, generated due to operating power sources and the resistive components of the sensor.

Here, intrinsic and extrinsic noise of the sensor can be modeled as:

(2) $$\left\{\begin{array}{c} V_{int}{}^{2}\approx V_{thermal}{}^{2}=4k_{B}TR_{⊥}\\ V_{ext}{}^{2}=V_{sys}{}^{2}+V_{env}{}^{2}\;\;\;\;\;\; \end{array}\right.$$

where *k_B_*, *T*, $R_{⊥}$, *V_sys_*, and *V_env_* refer to Boltzmann’s constant, absolute temperature, the sensor’s resistive component perpendicular to current direction, system noise contribution, and contribution from surrounding environment noise, respectively. Equation (2) reveals that thermal noise contribution can be estimated easily from the sensor’s resistive nature, whereas extrinsic noise components can only be analyzed through measurements. Moreover, it was found that system noise and environmental noise components can be identified from noise measurements, and their individual contribution can be estimated from NSD analysis. On the other hand, intermixing noise can be reckoned through noise measurements of the operating electronics sources and the associated resistive components. In order to correlate the sensor parameter and operating system noise, it is required to find the resistance, current and voltage relationship.

In a 2D self-balanced bridge configuration, Ohm’s Law for PHMR is given by:

(3) $$\left(\begin{array}{c} V_{x}\left(H\right)\\ V_{y}\left(H\right) \end{array}\right)=\left(\begin{array}{cc} R_{xx}\left(H\right) & R_{xy}\left(H\right)\\ R_{yx}\left(H\right) & R_{yy}\left(H\right) \end{array}\right)\left(\begin{array}{c} I_{x}\\ I_{y} \end{array}\right)$$

with

(4) $$\left\{\begin{array}{c} V_{x}\left(H\right)=I_{x}R_{xx}\left(H\right)+I_{y}R_{xy}\left(H\right)\\ V_{y}\left(H\right)=I_{x}R_{yx}\left(H\right)+I_{y}R_{yy}\left(H\right) \end{array}\right.$$

where $\left(\begin{array}{c} V_{x}\left(H\right)\\ V_{y}\left(H\right) \end{array}\right)$, $\left(\begin{array}{cc} R_{xx}\left(H\right) & R_{xy}\left(H\right)\\ R_{yx}\left(H\right) & R_{yy}\left(H\right) \end{array}\right)$ are MR voltage and apparent resistance tensor of the bridge structure. Note that both voltage components and resistance components show an applied field dependency. *I_x_*, *I_y_* refers here to operating current components along the x-axis and y-axis, whereas *V_x_*, *V_y_* represents output voltage along the x-axis and y-axis. In an ideal self-balanced bridge configuration, when *I_x_* ≠ 0 and *I_y_* = 0, *V_y_(H)* induced in y-direction corresponds to the sensor’s output signal. In ideal effective PHMR electrode configurations with *I_x_* ≠ 0 and *I_y_* = 0, PHMR voltage *V_y_* is given by:

(5) $$V_{y}\left(H\right)=R_{yx}\left(H\right)\left(I_{x}+\delta I_{n}\right)=I_{x}R_{yx}\left(H\right)\left(1+\frac{\delta I_{n}}{I_{x}}\right)=V_{y}\left(H\right)+V_{y}\left(H\right)\cdot \left(\frac{\delta I_{n}}{I_{x}}\right)$$

where $\delta I_{n}$ is Nyquist noise of the operating current from the power source. A voltage drop will occur along the y-axis due to a fabrication mismatch of the PHMR electrode arms, and corresponds to the sensor offset voltage. In this condition, both *V_y_* and *R_yx_* exhibit a nonzero value due to a fabrication error, even if *I_y_* = 0 (see Equation (4)), and intermixing noise in the sensor output is given by:

(6) $$V_{mix}=\delta V_{y}\left(H\right){|}_{H=0}=V_{y}\left(H\right)\left(\frac{\delta I_{x}}{I_{x}}\right){|}_{H=0}=V_{offset}\left(\frac{\delta I_{n}}{I_{x}}\right)$$

with

(7) $$V_{y}\left(H\right){|}_{H=0}\equiv V_{offset}$$

where *I_x_* and *R_yx_* are the sensor operating current and off-diagonal PHMR tensor component, respectively. Note that *V_offset_* represents the DC-offset voltage of the bridge sensor for H = 0 mT, and confirms a correlation with the operating current (see Equation (6)), which eventually generates the intermixing noise in the sensor.

### 3.2. Decomposition of Noise Components

**Figure 3:**
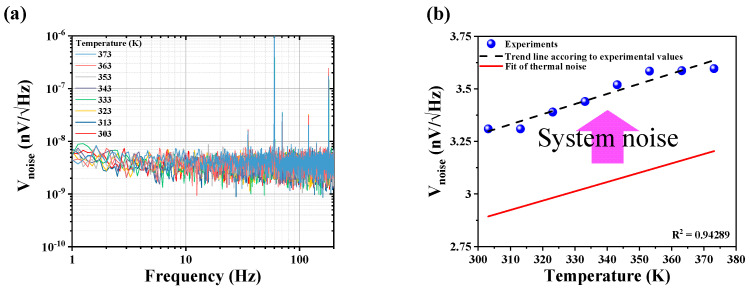
(**a**) Measurement of NSD for temperature of the PHMR sensor. The NSD was measured while changing the temperature at 10 K intervals from 303 K to 373 K. (**b**) Slopes of average noise measurement value for each temperature and thermal noise calculation value for each temperature were identical.

To investigate the thermal noise contribution explicitly, the measurements were performed inside a furnace to maintain a uniform temperature around the sensor in the range of 303 K~373 K with 10 K steps. As shown in Figure 3a, the change in NSD with temperature variation was nominal, and it was difficult to distinguish the different NSDs. To get an accurate comparison of different NSDs, we plotted the estimated average noise as a function of system temperature (see Figure 3b). The blue dot points correspond to experimentally obtained noise data points, whereas the solid black line refers to the linear fit model, as *V_noise_* was proportional to change in temperature. Importantly, the incremental change in the observed experimental data coincided with the theoretical estimation, and corroborated the validity of the system noise model. As mentioned above, the average noise was extracted from the NSDs at ~100 Hz, where white noise was more dominant. The solid red line indicates the variation of average estimated thermal noise with a y-directional resistive component of sensor (500 Ω). Figure 3b shows that the average noise increased gradually with the increase of temperature, and reached its maximum value of ~3.59 $\mathrm{nV}/\sqrt{\mathrm{Hz}}$ at 373 K. The estimated change in average noise was observed at ~0.28 $\mathrm{nV}/\sqrt{\mathrm{Hz}}$ starting from its room temperature counterpart; i.e., 3.31 $\mathrm{nV}/\sqrt{\mathrm{Hz}}$ at 303 K. However, with a 500 Ω sensor input resistance, the corresponding incremental change in average noise was found to be ~0.31 $\mathrm{nV}/\sqrt{\mathrm{Hz}}$. Importantly, the total changes in sensor noise as extracted from experimental and numerical resistive models were found very close to each other. This signature confirmed the predominant existence of thermal noise in total noise.

**Figure A1:**
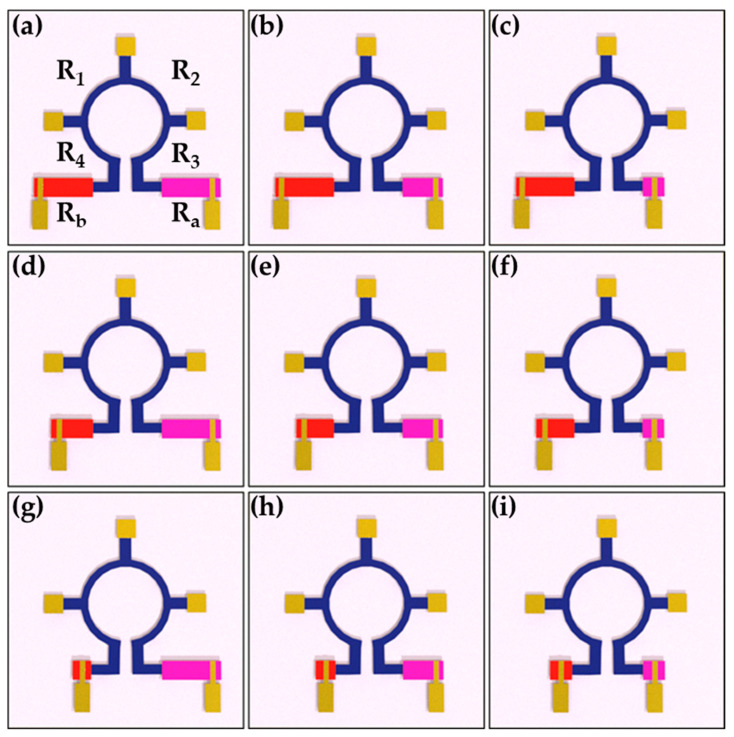
Example of various resistance-compensated self-balancing bridge-type PHMR sensors. Different compensators geometries have been configured based on their lengths: long, medium, and short and are shown in (**a**–**i**). Compensators were marked with Red and Purple colors.

Second, taking into account Equation (6), the noise on the self-balanced bridge is expected to be proportional to the magnitude of the offset voltage, thus we fabricated multiple PHMR sensors with different shape parameters (see Figure A1) to modulate the shape-induced offset voltages and investigated their impact of changes in NSDs. Note that the shape change of each sensor could be tuned geometrically using the branch length compensator of the electrodes, as shown in Figure A1. In the present case, the sensor shape architectures were reconfigured by increasing their unit length in both branches (left and right) by 35 μm and 70 μm, respectively. It was found that in the reconfigured sensor architecture with incremental change in the branch length of each arm, the corresponding offset voltage also increased gradually: 0.07 mV, 2.43 mV, and 6.53 mV, respectively. In the next step, we analyzed the measured NSDs for different offset voltages to find a correlation between sensor noise levels and offset voltages.

**Figure 4:**
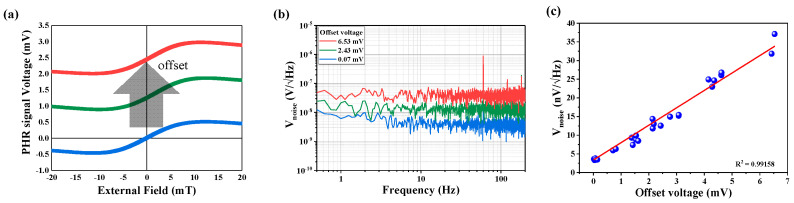
NSDs of the sensors were measured by tuning the balancing resistance of the compensator. (**a**) PHMR profile showing the different offset voltage that can be adjusted by resistance-regulated voltage. (**b**) Comparison of NSDs for different resistance tuner electrodes with offset voltage. (**c**) The blue dot is the value measured through the experiment, and the red line is the value obtained by linear fitting from the measured value.

Figure 4a shows the PHMR signal profile measurements of all sensors, which were performed in the field range of −20 mT to 20 mT using a Helmholtz coil with a step-size of 0.13 mT, and confirmed the PHMR antisymmetric nature and depicted the sensor linearity. The magnetic field was measured with a Lakeshore 450 Gauss meter at a field resolution of 0.001 mT. Furthermore, the output PHMR voltage of each sensor increased linearly with the applied field, irrespective of the offset, perpendicular to the easy magnetization axis, followed by a gradual dropdown after reaching its maximum magnetization angle value (π/4). This confirmed the conventional planar Hall characteristic of the PHMR sensors.

Figure 4b shows the enhancement in average noise from 3.8 $\mathrm{nV}/\sqrt{\mathrm{Hz}}$ to 37.1 $\mathrm{nV}/\sqrt{\mathrm{Hz}}$ with the increase of the offset voltage, as discussed above. In each case, the offset voltage error was adjusted using the offset resistance tuner as a compensator. Based on the change in device offset voltages, the obtained noise spectra for different resistance tuner electrodes with offset voltage are shown in Figure 4b. It was observed that with an increase of the system offset voltage, the baseline of the NSDs was shifted to the higher noise levels. To find a correlation between the change in offset voltage with NSDs, a multiple set of data was recorded for different offset voltages, and the noise mean values for each spectrum were calculated. The change in noise mean values with change in offset voltage are displayed in Figure 4c; it depicts that total average noise held a linear relationship with device offset voltage, and validates the proposed model as described in Equation (6). Note that similar measurements were carried out repeatedly to confirm the experimental results.

In addition, the fit equation as shown in Figure 4c can be expressed as follows:

(8) $$V_{noise}=a+b\cdot V_{offset}\;\left\{\begin{array}{c} a=3.34\times 10^{-9}\\ b=4.66\times 10^{-6} \end{array}\right.$$

It is evident that at zero offset voltage, Equation (8) only measures the total noise of the sensor, *V_noise_*, and gives a value of ~3.34 $\mathrm{nV}/\sqrt{\mathrm{Hz}}$. However, by subtracting the thermal noise (2.89 $\mathrm{nV}/\sqrt{\mathrm{Hz}}$) contribution, we can estimate the extrinsic noise contribution in the system, *V_ext_* = 1.67 $\mathrm{nV}/\sqrt{\mathrm{Hz}}$. Generally, extrinsic noise contribution comes from the power source, measuring equipment, and other external sources. It was found that the measuring equipment had an average noise of 1 $\mathrm{nV}/\sqrt{\mathrm{Hz}}$ by connecting the line itself without the sensor element. According to Equation (6), it was found that the intermixing noise was proportional to the sensor resistance and the operating current.

**Figure 5:**
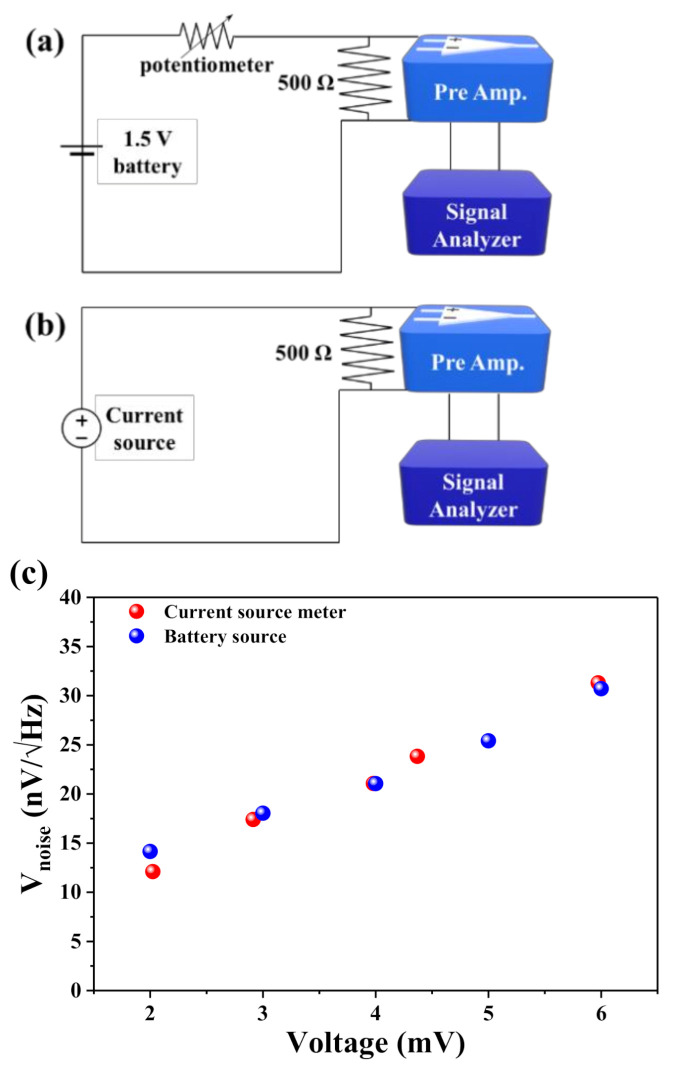
(**a**,**b**) Schematic diagrams of the device configurations for measuring NSDs of the 500 Ω resistor using both the battery module and current source meter. (**c**) Estimated average noise of the 500 Ω resistor for both measurement schemes.

In order to confirm the intermixing noise characteristics generated from the operating current sources, a current source meter and a 1.5 V battery were connected to a resistor of 500 Ω, and the corresponding NSDs were recorded for different bias voltages using a spectrum analyzer. The experimental setups for both modules are shown in Figure 5a,b, respectively. Figure 5c reveals that the total average noise increased linearly with the output voltage of both power sources (source meter and battery module).

**Figure 6:**
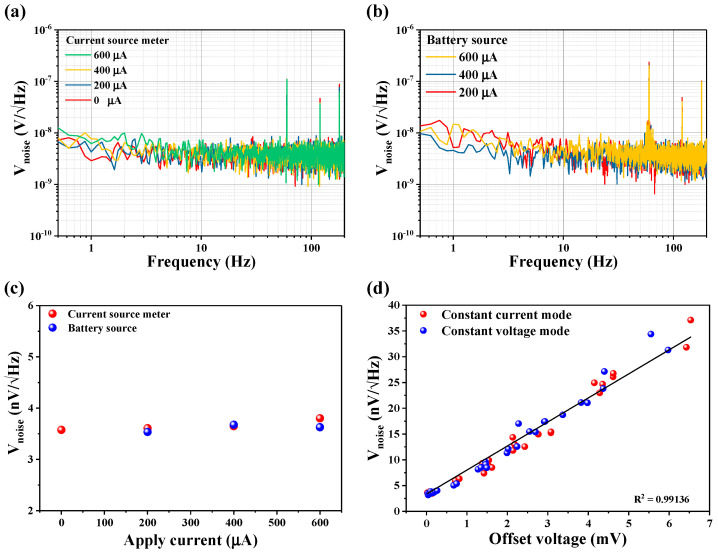
(**a**) NSD measurements using the driving current source for different driving currents; (**b**) NSD measurements using a battery source for different driving currents; (**c**) average noise comparison between the current source meter and battery source driving methods; (**d**) comparison of noise voltage in constant curent and voltage modes. Noise measurements were performed using a source meter. The solid black line shows the fit as depicted in Equation (8).

For an experimental demonstration, we first measured the difference in the recorded NSDs for both power sources mounted with the same sensor and then adjusted *R_yx_* ≈ 0. Figure 6a,b depict both device NSD increases in the frequency range between 0.5 Hz and 200 Hz with an increase of bias currents from 200 μA to 600 μA. The average noise is shown in Figure 6c. Note that the average noise values varied between 3.57 $\mathrm{nV}/\sqrt{\mathrm{Hz}}$ and 3.80 $\mathrm{nV}/\sqrt{\mathrm{Hz}}$ for the current source, whereas for the battery source, the deviation varied between 3.53 $\mathrm{nV}/\sqrt{\mathrm{Hz}}$ and 3.62 $\mathrm{nV}/\sqrt{\mathrm{Hz}}$. These results show that in both operating methods, average noise levels exhibited similar characteristics and showed a negligible increment with a sensor operating voltage/current, thus it depicted a minimal contribution in the total sensor noise. Therefore, power source noise may be ignored in the total sensor noise when *V_offset_* is set to zero; i.e., *R_yx_ =* 0. On the contrary, in the unbalanced bridge configuration (i.e., *R_yx_* ≠ 0), it was found that the average sensor noise linearly increased with offset voltage regardless of the amplitudes of biasing current or voltage-operated by a current source (see Figure 6d), and confirmed that the sensor noise was proportional to the offset voltage, as in Equation (8). Furthermore, extrinsic noise contributions could also be determined from the previously estimated thermal noise and Equation (8) relationship. It was found that for an extrinsic noise contribution of 1.67 $\mathrm{nV}/\sqrt{\mathrm{Hz}}$, a 1 $\mathrm{nV}/\sqrt{\mathrm{Hz}}$ noise contribution originates from the measurement device, whereas the rest of the noise contribution ~1.34 $\mathrm{nV}/\sqrt{\mathrm{Hz}}$ appeared from environmental sources. All noise components are summarized in Table 1.

## 4. Conclusions

In summary, the intrinsic sensor offset voltage was minimized in the reconfigured self-balanced bridge architecture by adjusting the unbalanced bridge resistance at the wafer level, which eventually improved the average noise level of the sensor. In addition, low-field noise measurements were performed using self-balanced bridge-type PHMR sensors for different operating bias currents and voltages. The contribution of thermal noise, extrinsic noise, and intermixing noise due to the change in offset voltages were examined and analyzed explicitly. Moreover, it was envisaged that the detectivity in such a self-balanced bridge-type sensor compensator could be improved dramatically due to suppression of the fabrication offset voltage error. In particular, the existing technologies, such as auto-zeroing and chopping, which were used to remove the offset voltage, required a complex circuit configuration, and additional noise was generated by circuits, including residual offset voltage and chopper ripple caused by a mismatch in capacitance. Furthermore, to highlight the strengths and drawbacks of existing offset removal technologies, such as auto-zeroing, chopping, and resistance compensator technologies, a comparative table was added (see Table 2) to better illustrate the advantages of the proposed design. Since the intermixing noise of the proposed sensor device was minimized, the noise level was substantially reduced from 10 $\mathrm{nV}/\sqrt{\mathrm{Hz}}$ to the level of thermal noise, thus the detectivity level was expected to be an order of magnitude or higher compared to any other type of similar magnetic sensors. We believe that they are promising candidates for future automotive, advanced electronic, and/or biosensing/biomedicine applications.

## Figures and Tables

**Table 1:** Kinds of noise: intrinsic, extrinsic, and intermixing noise.

Intrinsic Noise	Extrinsic Noise		Intermixing Noise
Thermal	Preamp.	Environ.	Coefficient *
$2.87\;\mathrm{nV}/\sqrt{\mathrm{Hz}}$	$1\;\mathrm{nV}/\sqrt{\mathrm{Hz}}$	$1.34\;\mathrm{nV}/\sqrt{\mathrm{Hz}}$	$4.66\times 10^{-6}$

* Coefficient of intermixing noise depends on the Nyquist noise of the operating current source.

**Table 2:** Offset cancellation techniques.

	Auto-Zeroing	Chopping	Resistance Compensator
Advantage	No loss of bandwidth Removes ripple Reduces drift	No effect on white noise Reduces drift	Independent of circuit bandwidth No calibration circuits Minimizes intermixing noise of the sensor itself Reduces drift
Disadvantage	Power noise efficiency High circuit complexity Residual offset voltage from capacitor Remaining intermixing noise	Loss of bandwidth High circuit complexity Continuous measurement Chopper ripple Remaining intermixing noise	Large sensor area Only wheatstone bridge

## Data Availability

All data generated or analyzed during this work are included in this article.

## Appendix A

In general, when an imbalance of $R_{1}$, $R_{2}$, $R_{3}$, and $R_{4}$ occurs in a self-balanced bridge, an offset voltage occurs. To adjust this imbalance, the offset voltage can be removed by adding adjustable resistors $R_{a}$ and $R_{b}$ as shown in Figure A1a, and adjusting the formula $R_{1}\times \left(R_{3}+R_{a}\right)=R_{2}\times \left(R_{4}+R_{b}\right)$ to be established. $R_{1}$, $R_{2}$, $R_{3}$, and $R_{4}$ are determined in the fabrication process, so the offset voltage can be adjusted by the resistance change of $R_{a}$ and $R_{b}$. The resistance of $R_{a}$ and $R_{b}$ depends on the combination of electrode connection, and the connection combination is expressed as shown in Figure A1. For example, based on Figure A1a, Figure A1b shows that the length of $R_{a}$ (purple bar) is 1/2, so the resistance decreases from 40 Ω to 20 Ω, and the offset decreases. Conversely, when it becomes (d), the resistance of $R_{b}$ (red bar) decreases from 80 Ω to 40 Ω, and the offset increases, and the offset of the PHMR sensor can be adjusted through this combination of electrodes. In addition, by adjusting branch resistance of the compensator, we experimentally implemented resistance compensation in units of 1 Ω, and this is expected to achieve lower level of resistance compensation in a similar approach with different low-resistive materials.
